# Electroacupuncture following microsurgical neurorrhaphy: functional and histological outcomes in a rat model

**DOI:** 10.1590/acb415726

**Published:** 2026-10-19

**Authors:** Felipe Giraldo Alvarez Gonçalves, Liaw Wen Chao, Emily Caroline da Fonseca Sampaio, Adriano Augusto Domingos, Luiza Rafih Abud, André Salo Buslik Hazan, Fausto Viterbo, Rolf Gemperli, Cristina Pires Camargo, Wu Tu Hsing

**Affiliations:** 1 Universidade de São Paulo – School of Medicine – Department of Surgery – São Paulo (SP) – Brazil.; 2 Universidade de São Paulo – School of Medicine – Laboratory of Plastic Surgery and Regenerative Medicine – São Paulo (SP) – Brazil.; 3 Universidade de São Paulo – Center of Acupuncture – Department of Orthopedics and Traumatology – São Paulo (SP) – Brazil.; 4 Sociedade Brasileira de Microcirurgia Reconstrutiva – Rio de Janeiro (RJ) – Brazil.; 5 Asociación Latinoamericana de Microcirurgía – Rio de Janeiro (RJ) – Brazil.; 6 Universidade Estadual Paulista – School of Medicine – Department of Plastic Surgery – Botucatu (SP) – Brazil.

**Keywords:** Peripheral Nerves, Peripheral Nerve Injuries, Electroacupuncture, Acupuncture

## Abstract

**Purpose:**

This study evaluated electroacupuncture (EA) as adjunctive therapy after peripheral nerve repair in a rat model.

**Methods:**

Male Wistar rats underwent right sciatic nerve transection followed by neurorrhaphy and were randomized into EA or control groups. EA was performed twice weekly for 12 weeks. Sensory and motor recovery were assessed by nociceptive, mechanical, thermal, and sciatic functional index tests. Histological analyses evaluated Schwann cell and vascular densities, and gastrocnemius muscle weight was measured at euthanasia.

**Results:**

Sensory recovery was significantly greater in the EA group, with higher pain (3 [3–3.75] *versus* 2 [1–2], *p* = 0.02), pressure (2.5 [1.25–3] *versus* 0 [0–1], *p* = 0.009), and thermal sensitivity scores (2 [2–2.75] *versus* 0 [0–0], *p* = 0.02). Motor recovery and vascular density did not differ between groups. Distal Schwann cell density was significantly higher in the EA group (73.5 [67.4–74.1] *versus* 34.7 [34.5–38.4], *p* = 0.014). Gastrocnemius muscle weight was reduced in operated limbs in both groups, without intergroup differences.

**Conclusion:**

EA improved sensory recovery and increased distal Schwann cell density, supporting its potential as an adjunctive therapy after peripheral nerve repair.

## Introduction

Peripheral nerve injury (PNI) is a significant cause of long-term disability in trauma care, affecting approximately 5% of injured patients^1,2^. Common mechanisms include laceration, traction, ischemia, compression, and electrical burns^3,4^. Although advances in microsurgical techniques have improved nerve repair, many patients still experience incomplete recovery of motor, sensory, or autonomic function^5,6^. These limitations reflect the intrinsic biological constraints of peripheral nerve regeneration.

Axonal regeneration occurs slowly, at approximately 1 mm per day, and prolonged denervation can lead to irreversible degeneration of neuromuscular junctions, muscle atrophy, and replacement by fibrotic tissue^3,7^. Although microsurgical neurorrhaphy remains the gold standard for the treatment of nerve transections, functional recovery is often incomplete, particularly when reinnervation is delayed^8^. Consequently, strategies aimed at enhancing axonal regeneration and improving functional outcomes have gained increasing attention.

Electrical stimulation has shown promising results in experimental models, accelerating axonal growth and promoting the expression of neurotrophic factors^9^. Electroacupuncture (EA) represents a specific modality of electrical stimulation delivered through acupuncture needles and has been suggested to modulate neural regeneration pathways. However, its potential role as an adjuvant therapy for peripheral nerve repair remains poorly defined. Therefore, this study aimed to evaluate the effects of EA as an adjuvant treatment for peripheral nerve repair.

## Methods

All experimental procedures were approved by the Institutional Animal Care and Use Committee of the Universidade de São Paulo School of Medicine (protocol no. 1,720/2021). The study was conducted in accordance with the Guide for the Care and Use of Laboratory Animals and reported in compliance with the Animal Research: Reporting of In Vivo Experiments (ARRIVE) guidelines^10^.

Male Wistar rats aged 8 weeks and weighing 200–250 g were used. Animals were housed in the institutional animal facility under controlled environmental conditions, with free access to food and water (*ad libitum*) and a 12:12-hour light–dark cycle.

### Surgical procedure

Under general inhalation anesthesia (2% isoflurane), the inguinal region was shaved and disinfected with 0.3% chlorhexidine solution (Rioquímica, Brazil). A skin incision was made at the posterior aspect of the right proximal thigh, followed by blunt dissection of the gluteal and biceps femoris muscles to expose the right sciatic nerve. The nerve was transected immediately proximal to its bifurcation. A tension-free end-to-end neurorrhaphy was then performed with four stitches between the proximal and distal stumps using 10-0 monofilament nylon sutures (Ethicon^®^, Johnson & Johnson, NJ, United States of America) under 10× microscopic magnification (ZEISS^TM^, Germany). The use of four stitches followed an institutional protocol established in a preliminary pilot study (unpublished data) using 8-week-old rats with body size and weight comparable to those included in the present study, in which this configuration provided satisfactory nerve-stump coaptation. After repair, layered closure was completed with interrupted 4-0 polyamide sutures (Ethicon®, Johnson & Johnson, NJ, United States of America). Sutures were removed on postoperative day 7. Animals were subsequently randomized into one out of two experimental groups: EA or control.

During the postoperative period, animals wore protective head cones to prevent biting and autotomy of the operated limb. Animals were monitored for self-directed biting, digit injury, and other postoperative complications.

### Electroacupuncture

EA was performed exclusively in the EA group using an EL30 Finder^®^ electrostimulator and locator (NKL Produtos Eletrônicos, Brusque, SC, Brazil) equipped with a dual-detection probe to identify areas of lower impedance and facilitate current delivery. Needle stimulation was delivered using an EL608 V2 stimulator (NKL Produtos Eletrônicos, Brusque, SC, Brazil), programmed to generate non-polarized pulsed electrical current with low-frequency parameters ranging from 2 to 10 Hz (EA-FR mode) and a pulse width of 250 µs. This mode delivers a pseudo-random sequence of frequencies within the 2–10 Hz range. Current intensity was individually adjusted to the lowest level capable of eliciting a visible motor response (0.1–2 mA).

EA was performed exclusively in the EA group using an EL30 Finder^®^ electrostimulator and locator (NKL Produtos Eletrônicos, Brusque, SC, Brazil) equipped with a dual-detection probe to identify areas of lower impedance and facilitate current delivery. The stimulation parameters and anatomical targeting procedure were established in a preliminary pilot study conducted in our laboratory (unpublished data). Needle placement was performed in the posterolateral aspect of the distal thigh, approximately 0.7 cm proximal to the knee joint. At each session, the precise stimulation point within this predefined anatomical region was identified as the site of the lowest electrical impedance detected by the EL30 Finder®. Needle stimulation was delivered using an EL608 V2 stimulator (NKL Produtos Eletrônicos, Brusque, SC, Brazil), programmed to generate non-polarized pulsed electrical current with low-frequency parameters ranging from 2 to 10 Hz (EA-FR mode) and a pulse width of 250 µs. This mode delivers a pseudo-random sequence of frequencies within the 2–10 Hz range. This low-frequency pseudo-random stimulation pattern was selected based on the pilot study to avoid continuous stimulation at a single fixed frequency while maintaining consistent motor activation. Current intensity was individually adjusted to the lowest level capable of producing visible, repetitive, and vigorous contractions of the operated limb, which served as functional confirmation of effective stimulation (0.1–2 mA).

EA sessions were initiated on postoperative day 7 and performed twice weekly for 20 minutes in the operated limbs of animals allocated to the EA group until the end of the 12-week postoperative period.

### Sensory function analysis

Sensory recovery was assessed through behavioral tests evaluating responses to nociceptive, mechanical, and thermal stimuli applied to the plantar surface of the operated hind limb. Pain sensitivity was evaluated by digital pressure applied to the plantar surface of the paw. Mechanical sensitivity was assessed using nylon monofilaments of different calibers applied perpendicularly to the plantar surface to determine the withdrawal response. Thermal sensitivity was evaluated using a hot plate maintained at 55°C, and the withdrawal response of the hind paw was recorded. Responses were graded using a semi-quantitative scale ranging from 0 to 4, where 0 indicated absence of response and 4 indicated a normal withdrawal response comparable to the contralateral limb. A single assessment was performed per animal for each sensory outcome, yielding one integer score ranging from 0 to 4. All evaluations were performed by the same blinded examiner at the end of the follow-up period (week 12).

### Motor function analysis

Motor function evaluation of sciatic nerve regeneration in rats was performed using the walking track (WT) method, which assesses gait alterations resulting from neural injury leading to gait alteration in the operated limb. Gait analysis was performed at the following time points: preoperative, and postoperative at the third, sixth, ninth, and 12th weeks.

The WT apparatus was made of wood, measuring 13 cm in width and 73 cm in length. A sheet of white paper of the same dimensions was placed on the corridor floor before placing the animal at one end. Immediately before the test, the hind paws of the rats were coated with black India ink, and the animals were allowed to walk freely along the track, leaving footprints on the paper. Prior to any surgical procedure, rats were trained to walk along the wooden testing corridor five times (back and forth) to ensure familiarity with the setup and facilitate spontaneous ambulation during testing.

All measurements were performed by the same examiner across the two experimental groups and at all study time points, who was blinded to group allocation. The following parameters of the hind paw prints were recorded:

- Print length (PL), defined as the distance from the tip of the third toe to the heel;
- Toe spread (TS), defined as the distance between the first and fifth toes;
- Intermediary toe spread (IT), defined as the distance between the second and fourth toes.

These variables were used to calculate the sciatic functional index (SFI) according to the Bain–Mackinnon–Hunter formula^11^ (Eq. 1):


(1) $$\mathrm{SFI}=-38.3\left(\frac{\mathrm{EPL}-\mathrm{NPL}}{\mathrm{NPL}}\right)+109.5\left(\frac{\mathrm{ETS}-\mathrm{NTS}}{\mathrm{NTS}}\right)+13.3\left(\frac{\mathrm{EIT}-\mathrm{NIT}}{\mathrm{NIT}}\right)-8.8$$

where: EPL: experimental print length; NPL: normal print length; ETS: experimental toe spread; NTS: normal toe spread; EIT: experimental intermediary toe spread; NIT: normal intermediary toe spread.

Values obtained from this formula were interpreted as indicators of sciatic nerve functional status. An SFI value close to 0 corresponds to normal nerve function, whereas values approaching -100 indicate complete functional impairment. Intermediate values reflect partial functional deficits.

### Gastrocnemius muscle atrophy assessment

At the 12th postoperative week, after euthanasia, the gastrocnemius muscles were harvested bilaterally from the operated limb (right) and the contralateral limb (left), which served as an internal control.

The posterior compartment of the hind limb was exposed, and the gastrocnemius muscle was dissected at its proximal insertion on the femoral condyles and distal insertion on the calcaneus. The harvested muscles were then weighed using a high-precision digital scale (Precision PR-100, 110/220 volts, Dourado, Brazil).

### Sciatic nerve harvesting

At the 12th postoperative week, after euthanasia, an incision was made in the posterior region of the right thigh, following the same approach used during the initial sciatic nerve resection and repair surgery. An 8-mm segment of the sciatic nerve, including both proximal and distal neurorrhaphy sites, was excised.

### Microscopic analysis

Following euthanasia, a 5-mm segment of the sciatic nerve including the neurorrhaphy site was harvested. Histological sections were obtained at three standardized locations: 2 mm proximal to the neurorrhaphy site, at the neurorrhaphy site itself, and 2 mm distal to it. Specimens were fixed in 10% formalin for up to 72 hours, embedded in paraffin, and sectioned into 1-µm-thick slices.

Histological sections were stained with hematoxylin–eosin (HE) and Masson’s trichrome (MT) to evaluate general morphology and connective tissue organization. Analyses focused on Schwann cell distribution (Fig. 1) and vascular density. All evaluations were performed by a blinded investigator using a Nikon E200 microscope (Nikon, United States of America) at 40× magnification. Ten representative microscopic fields were analyzed per specimen for histomorphometric assessment, and the mean value was used for statistical analysis.

**Figure 1:**
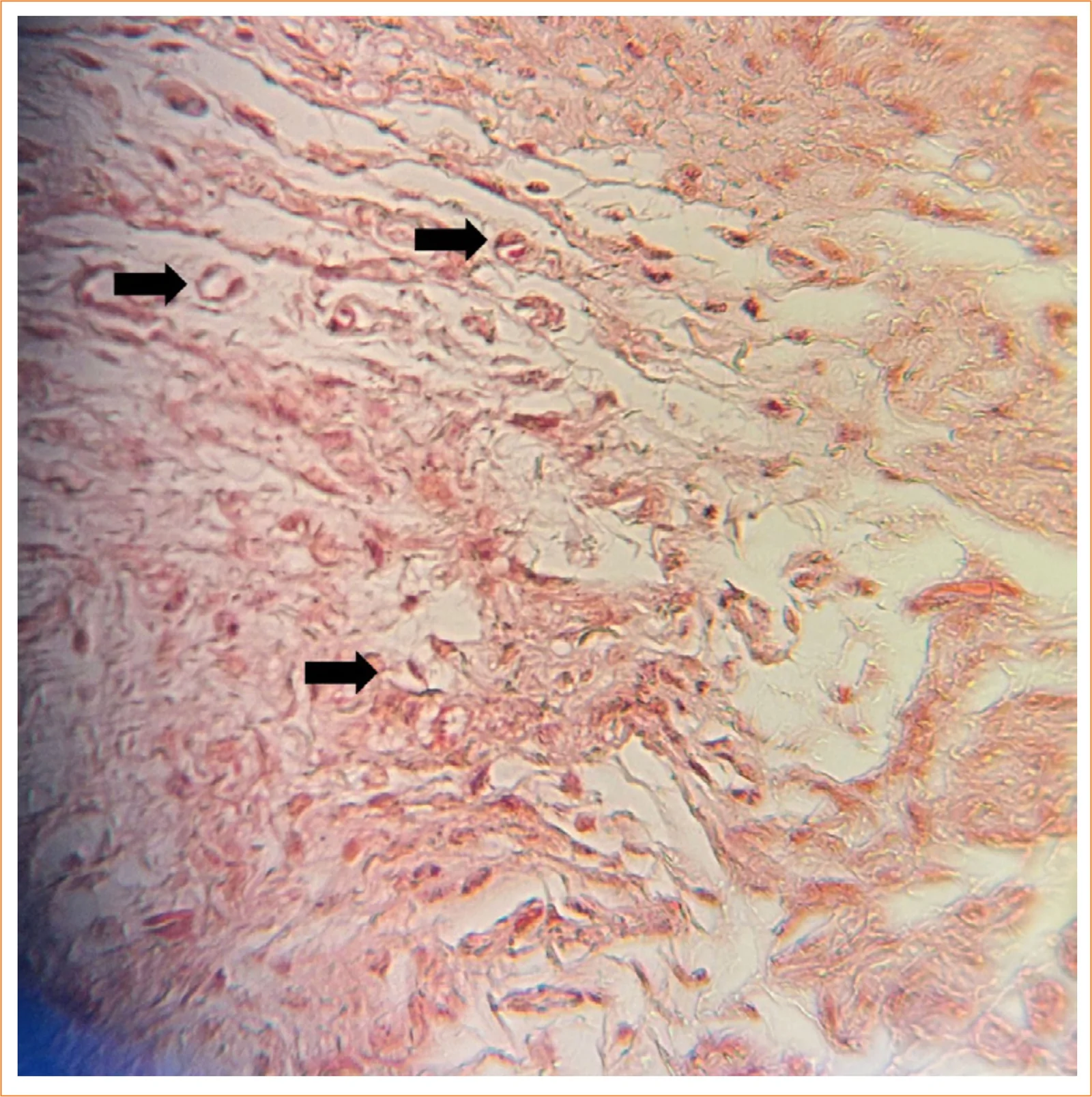
Histological image illustrating Schwann cell identification. Masson’s trichrome staining at 40× magnification. Schwann cells were identified based on their characteristic elongated, peripherally located nuclei associated with nerve fibers. Black arrows indicate representative Schwann cells.

SFI: sciatic functional index; SD: standard deviation; EA: electroacupuncture. Source: Elaborated by the authors.

### Endpoints

The primary endpoints of the study were sensory recovery evaluated through nociceptive, mechanical, and thermal response tests and motor functional recovery assessed by the SFI. Secondary endpoints included histological assessment of Schwann cell density and vascular density in proximal, intermediate, and distal nerve segments, as well as gastrocnemius muscle weight.

### Statistical analysis

#### Sample size calculation

Sample size was calculated assuming an alpha of 0.05, study power of 80%, and an expected clinically relevant difference of 0.45 between groups, with an estimated standard deviation of 0.20. Under these parameters, a minimum of five animals per group was required. These values are consistent with previously reported effect sizes in peripheral nerve regeneration models. To account for potential experimental losses, seven animals were included in each group.

#### Data analysis

Collected data were described according to the type and distribution of the variables. SFI values were summarized as mean ± standard deviation, whereas continuous and ordinal variables from the remaining outcomes were summarized as median and interquartile range (IQR). Motor functional recovery assessed by the SFI was analyzed using a linear mixed-effects model with group, time, and their interaction as fixed effects and animal as a random effect to account for repeated measurements. Post hoc pairwise comparisons were performed using Bonferroni adjustment. Sensory outcomes (pain, pressure, and thermal responses) were analyzed as ordinal variables and compared between groups using the Mann–Whitney’s U test. Histological outcomes, including Schwann cell counts and vascular density, were also compared between groups using the Mann–Whitney’s U test. Gastrocnemius muscle weight was compared among groups using the Kruskal–Wallis’ test, followed by Dunn’s post hoc test with Bonferroni correction.

A two-tailed significance level of 5% was adopted for inferential analyses. Statistical analyses were performed using R statistical software (R Foundation for Statistical Computing, Vienna, Austria).

Animals that died before completion of the experimental protocol or could not undergo outcome assessment were excluded from the final analysis.

## Results

### Animal loss

One animal in the EA group died on the first postoperative day and was excluded from the final analysis. Final group sizes were seven animals in the control group and six in the EA group, which remained above the minimum sample size required by the *a priori* power calculation. No episodes of autotomy or other postoperative complications occurred among the surviving animals.

### Sensory function

Sensory recovery was significantly greater in the EA group compared with control group. Pain scores were higher in the EA group than in control group (3 [3–3.75] *versus* 2 [1–2], *p* = 0.02). Similarly, pressure sensitivity scores were significantly higher in the EA group (2.5 [1.25–3] *versus* 0 [0–1], *p* = 0.009). Thermal sensitivity scores also demonstrated significantly greater recovery in the EA group compared with control group (2 [2–2.75] *versus* 0 [0–0], *p* = 0.02) (Table 1).

**Table 1:** Sensory outcomes at postoperative week 12 according to experimental group. Pain, mechanical pressure, and thermal responses were graded using a semiquantitative ordinal scale ranging from 0, indicating absence of response, to 4, indicating a normal withdrawal response comparable to the contralateral limb. A single assessment was performed per animal for each outcome. Values are presented as median (interquartile range) for control group (n = 7) and EA group (EA; n = 6). Between-group comparisons were performed using the Mann–Whitney’s U test.

Outcome	Control (n = 7)	EA (n = 6)	*p* -value
Pain	2 (1–2)	3 (3–3.75)	0.02
Pressure	0 (0–1)	2.5 (1.25–3)	0.009
Thermal	0 (0–0)	2 (2–2.75)	0.02

EA: electroacupuncture. Source: Elaborated by the authors.

### Motor function

No significant effects were observed for group (p = 0.18), time (p = 0.36), or the group-by-time interaction (p = 0.83), indicating similar patterns of motor recovery between the EA and control groups throughout the follow-up period. Mean SFI values remained comparable between groups at all evaluated time points for the control and EA groups, respectively (Fig. 2):

**Figure 2:**
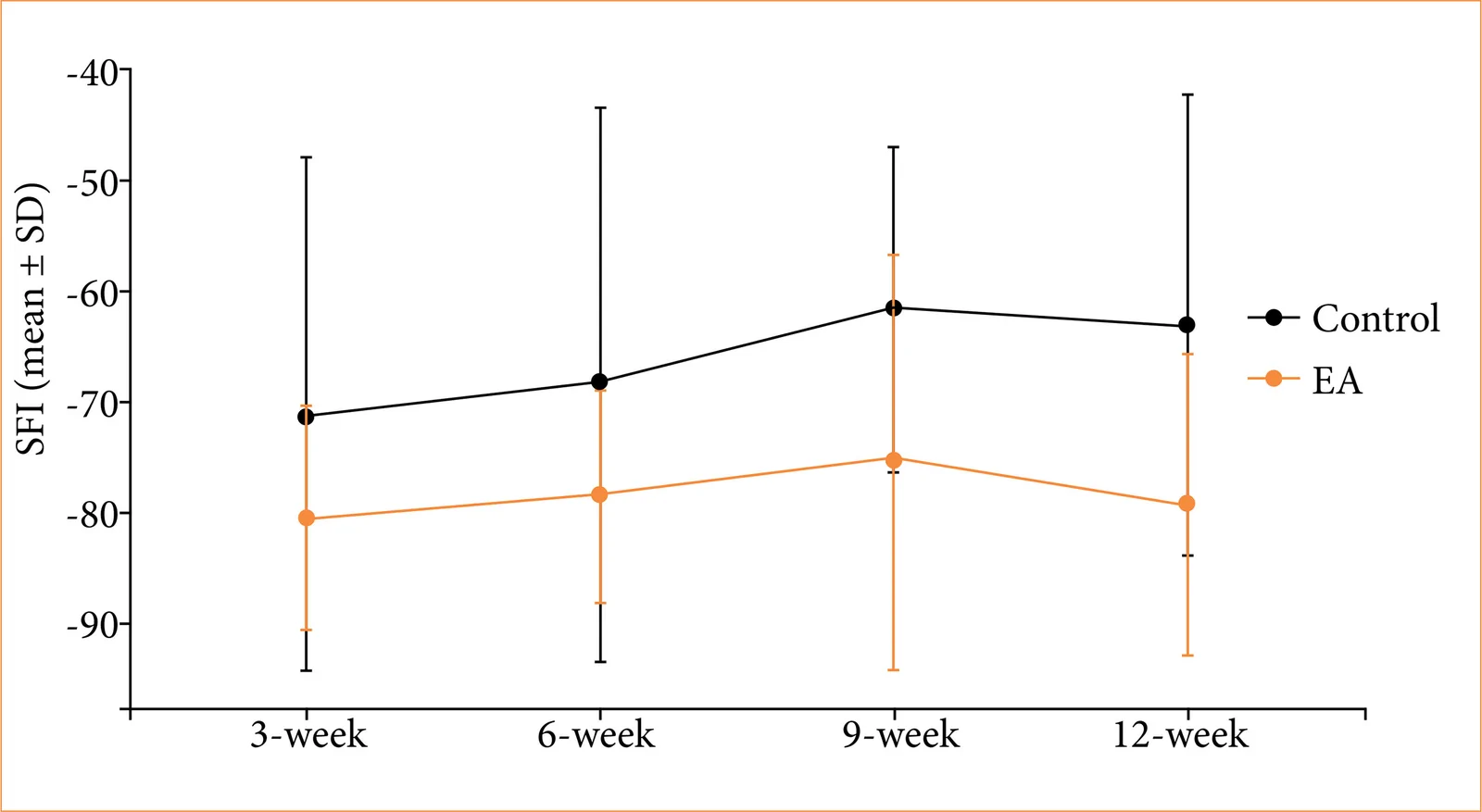
Sciatic functional index over time according to experimental group. Values represent the mean ± standard deviation of the sciatic functional index calculated for each animal from walking-track measurements using the Bain–Mackinnon–Hunter formula at postoperative weeks 3, 6, 9, and 12. Control group comprised seven animals, and the electroacupuncture group comprised six animals. More negative sciatic functional index values indicate greater functional impairment, whereas values closer to 0 indicate better functional recovery.

SFI: sciatic functional index; SD: standard deviation; EA: electroacupuncture. Source: Elaborated by the authors.

- Three weeks: -71.2 ± 23.2 versus -80.5 ± 10.2;
- Six weeks: -68.5 ± 25.1 versus -78.3 ± 9.6;
- Nine weeks: -61.5 ± 14.5 versus -75.4 ± 18.7;
- 12 weeks: -63.1 ± 20.9 versus -79.2 ± 13.6.

### Histological analysis

Histological analysis demonstrated similar vascular densities between groups in all evaluated nerve segments. Median (IQR) vessel counts in the proximal segment were 12.2 (9.7–13.2) in the control group and 9.7 (6.0–13.5) in the EA group (*p* = 0.57). In the intermediate segment, values were 13.4 (10.0–14.8) and 10.4 (6.1–17.3), respectively (*p* = 0.84). Distal segment vessel counts were also comparable between groups (15.5 [10.9–20.9] *versus* 16.2 [10.0–21.9], *p* = 0.93).

Schwann cell analysis demonstrated no significant differences between groups in the proximal segment (25.4 [24.2–33.8] *versus* 35.5 [28.3–38.5], *p* = 0.52) or in the intermediate segment (35.5 [27.6–44.0] *versus* 52.8 [41.8–59.0], *p* = 0.083). In contrast, the distal segment showed significantly higher Schwann cell counts in the EA group compared with control (73.5 [67.4–74.1] *versus* 34.7 [34.5–38.4], *p* = 0.014). Detailed histological results are presented in Table 2.

**Table 2:** Histological outcomes according to nerve segment and experimental group. Vascular and Schwann cell counts were evaluated in the proximal, intermediate, and distal sciatic nerve segments. For each animal and segment, 10 representative microscopic fields were analyzed at 40× magnification, and their mean was used as the individual value for statistical analysis. Group values are presented as median (interquartile range) for control group (n = 7) and electroacupuncture group (n = 6). Between-group comparisons were performed using the Mann–Whitney’s U test.

Segment	Vessels – control	Vessels – EA	*p* -value	Schwann cells – control	Schwann cells – EA	*p* -value
Proximal	12.2 (9.7–13.2)	9.7 (6.0–13.5)	0.57	25.4 (24.2–33.8)	35.5 (28.3–38.5)	0.52
Intermediate	13.4 (10.0–14.8)	10.4 (6.1–17.3)	0.84	35.5 (27.6–44.0)	52.8 (41.8–59.0)	0.083
Distal	15.5 (10.9–20.9)	16.2 (10.0–21.9)	0.93	34.7 (34.5–38.4)	73.5 (67.4–74.1)	**0.014**

EA: electroacupuncture. Source: Elaborated by the authors.

### Gastrocnemius muscle weight

Gastrocnemius muscle weight differed significantly among groups (Kruskal–Wallis’ test, χ^2^ = 19.84, *p* < 0.001). Median (IQR) gastrocnemius weights were 2.48 g (2.37–2.78) in the non-operated group, 1.50 g (1.14–1.60) in control group, and 1.12 g (0.94–1.40) in the EA group. Post hoc analysis using Dunn’s test with Bonferroni correction demonstrated that both operated groups had significantly lower muscle weight compared with the non-operated group (control group versus non-operated, *p* = 0.003; EA group *versus* non-operated, *p* < 0.001). No significant difference was observed between control and EA groups (i = 0.81).

## Discussion

PNIs represent a significant clinical and socioeconomic burden, with hospitalization costs reaching up to USD 64,000 per admission^12^. Functional recovery following PNI is often limited due to the inherently slow rate of axonal regeneration, estimated at approximately 1 mm per day^3,7^, as well as the molecular and structural changes associated with Wallerian degeneration in the distal nerve stump^13^. As a result, strategies aimed at enhancing nerve regeneration and improving functional recovery have been widely investigated^14^. Among them, EA has emerged as a potential therapeutic approach and was evaluated in the present study.

In the present study, EA significantly improved sensory recovery but did not produce measurable differences in motor function as assessed by the SFI. This discrepancy may reflect the different biological and functional requirements for sensory and motor recovery following PNI^15^. Sensory fibers are known to respond more rapidly to regenerative signals and neurotrophic stimulation, whereas motor recovery requires not only axonal regeneration but also appropriate guidance of motor fibers, remyelination, restoration of nerve conduction, and successful reinnervation of functional neuromuscular junctions^16^.

Therefore, the increased distal Schwann cell density observed in the EA group may reflect a more permissive regenerative microenvironment without necessarily indicating that a sufficient number of motor axons reached the target muscle and restored coordinated muscle activity. Moreover, Schwann cell density alone does not characterize their functional phenotype, their association with regenerating motor axons, or the extent of effective motor reinnervation. Because denervation-induced muscle atrophy begins early after nerve injury, through reduced protein synthesis, increased proteolysis, and progressive deterioration of the neuromuscular apparatus, a localized or delayed improvement in the distal nerve environment may have been insufficient to preserve muscle mass before functional reinnervation occurred.

Moreover, functional motor indices such as the SFI depend on complex biomechanical parameters and may be less sensitive to early regenerative changes^11^. Therefore, the improved sensory outcomes observed in the EA group may represent an early regenerative effect that precedes detectable improvements in motor function. Alternatively, the magnitude of the biological effect induced by EA may have been insufficient to overcome the multiple downstream requirements for motor recovery and prevention of denervation-induced atrophy within the 12-week study period.

Histological analysis demonstrated a significantly higher Schwann cell density in the distal nerve segment of animals treated with EA compared with control group. Schwann cells play a central role in peripheral nerve regeneration^17^. Following PNI, these cells proliferate and align longitudinally within the endoneurial tubes to form the bands of Büngner, which provide structural guidance for regenerating axons toward their distal targets^18^. The increased Schwann cell density observed distally in the EA group may indicate a more favorable regenerative microenvironment in the distal stump. In contrast, vascular density did not differ significantly between groups in any of the analyzed nerve segments, suggesting that the observed functional differences may be more closely related to Schwann cell dynamics rather than changes in vascularization.

This difference was limited to the distal segment, with no significant changes observed in the proximal or intermediate portions of the nerve. The distal stump is known to represent the most biologically active compartment during peripheral nerve regeneration, largely due to the molecular events associated with Wallerian degeneration^17^. In this region, Schwann cells and infiltrating cells upregulate neurotrophic factors that promote axonal growth and Schwann cell activation^19^. This biological characteristic may partially explain why the effect observed in the present study was restricted to the distal segment.

Experimental studies have suggested that EA may increase the expression of neurotrophic factors such as nerve growth factor (NGF) and brain-derived neurotrophic factor (BDNF), which can stimulate Schwann cell proliferation and support axonal regeneration^20^. More specifically, experimental evidence suggests that EA may regulate the miR-1b/BDNF axis, with reduced miR-1b expression allowing increased BDNF expression and subsequently promoting Schwann cell proliferation and migration^21^.

EA has also been associated with the regulation of exosome-mediated miR-21 signaling, through which Schwann cell-derived exosomes may transfer miR-21 to regenerating axons and promote neurite outgrowth21. In addition, EA-induced increases in NGF expression may further support Schwann cell proliferation, while modulation of GDNF and N-cadherin expression and attenuation of neuronal apoptosis have been described in other experimental models of PNI^22,23^.

Together, these findings suggest that the effects of EA unlikely depend on a single neurotrophic factor but may instead involve coordinated regulation of neurotrophic, microRNA-mediated, exosomal, and cell-survival pathways. Activated Schwann cells may in turn release additional neurotrophic factors, contributing to a regenerative feedback process^19^. Although neurotrophic signaling was not directly evaluated in the present study, those mechanisms may represent a plausible explanation for the increased Schwann cell density observed in the distal nerve segment and the improved sensory recovery detected in the EA group, suggesting that EA may promote a biologically favorable microenvironment for peripheral nerve regeneration. Nevertheless, because neurotrophic factor expression, microRNA regulation, and intracellular signaling were not directly assessed, these proposed mechanisms remain hypothetical in the context of the present study.

For the analysis of gastrocnemius muscle weight, the two experimental groups were included (EA and control), and the contralateral non-operated limb of each animal was considered as a reference group for this endpoint. Gastrocnemius muscle weight was significantly lower in the operated limbs compared with the contralateral non-operated limbs, reflecting the expected muscle atrophy that occurs following PNI and denervation^24^. Both operated groups showed significantly lower muscle weight compared with the non-operated limbs, while no significant difference was observed between the EA and control groups. Although EA involves electrical stimulation and could theoretically contribute to muscle preservation^25^, this effect was not observed in the present study. One possible explanation is that muscle atrophy following PNI is primarily driven by the loss of motor innervation and impaired neuromuscular transmission^24^. Therefore, in the absence of significant improvements in motor recovery, as reflected by the similar SFI values between groups, EA alone may not have been sufficient to prevent denervation-induced muscle atrophy.

This study has some limitations that should be considered when interpreting its findings. First, as an experimental study conducted in a rat model, the results may not fully reflect the complexity of human PNIs. Nevertheless, animal models remain an essential step for understanding regenerative mechanisms and testing potential therapeutic strategies. Second, although the sample size was consistent with previous experimental studies in peripheral nerve regeneration, larger cohorts could provide additional statistical robustness. Some of the functional assessments, particularly sensory testing and WT analysis, are also inherently operator-dependent, although all measurements were performed by the same blinded evaluator to minimize variability.

In addition, Schwann cell quantification was based on morphological assessment using MT staining, following a previously established and institutionally validated protocol^26^, without confirmation using Schwann cell-specific immunohistochemical markers such as S-100 protein. Therefore, these findings should be interpreted considering the lower cellular specificity of conventional histological staining.

Finally, the present study focused on functional and histological outcomes and did not include molecular analyses of neurotrophic factors or gene expression. Future investigations from our research group will aim to explore the biological mechanisms underlying these findings, including the evaluation of neurotrophic signaling and the potential synergistic effects of EA combined with adipose-derived stem cells.

From a translational perspective, EA may represent a feasible nonpharmacological adjunct after peripheral nerve repair, potentially initiated during the early postoperative rehabilitation period once wound healing permits. However, the optimal timing, session frequency, treatment duration, and stimulation parameters in humans remain undefined and should be established in prospective clinical studies before routine implementation.

## Conclusion

EA following microsurgical peripheral nerve repair improved sensory recovery and was associated with increased Schwann cell density in the distal nerve segment in this rat model. However, no significant effects were observed on motor functional recovery, muscle atrophy or vascular density. These findings suggest that EA may represent a potential adjunctive approach to enhance sensory recovery following peripheral nerve repair.

## Data Availability

Data are available from the corresponding author upon reasonable request.
